# Circadian clocks and neurodegenerative diseases: time to aggregate?^☆^

**DOI:** 10.1016/j.conb.2013.05.004

**Published:** 2013-10

**Authors:** Michael H Hastings, Michel Goedert

**Affiliations:** MRC Laboratory of Molecular Biology, Francis Crick Avenue, Cambridge Biomedical Campus, Cambridge CB2 0QH, UK

## Abstract

The major neurodegenerative diseases are characterised by a disabling loss of the daily pattern of sleep and wakefulness, which may be reflective of a compromise to the underlying circadian clock that times the sleep cycle. At a molecular level, the canonical property of neurodegenerative diseases is aberrant aggregation of otherwise soluble neuronal proteins. They can thus be viewed as disturbances of proteostasis, raising the question whether the two features — altered daily rhythms and molecular aggregation — are related. Recent discoveries have highlighted the fundamental contribution of circadian clocks to the correct ordering of daily cellular metabolic cycles, imposing on peripheral organs such as the liver a strict programme that alternates between anabolic and catabolic states. The discovery that circadian mechanisms are active in local brain regions suggests that they may impinge upon physiological and pathological elements that influence pro-neurodegenerative aggregation. This review explores how introducing the dimension of circadian time and the circadian clock might refine the analysis of aberrant aggregation, thus expanding our perspective on the cell biology common to neurodegenerative diseases.

## Highlights

• Neurodegenerative diseases are characterised by disordered sleep/wake patterns.
• Aggregation of soluble proteins is the canonical molecular motif of neurodegeneration.
• Local circadian clocks direct processes influencing pro-neurodegenerative aggregation.
• Thus, circadian/sleep–wake timing and neurodegeneration are reciprocally dependent.
• Revealing the feedback between clocks and neurodegeneration may suggest new therapies.

**Current Opinion in Neurobiology** 2013, **23**:880–887

This review comes from a themed issue on **Circadian rhythm and sleep**

Edited by **Clifford Saper** and **Amita Sehgal**

For a complete overview see the Issue and the Editorial

Available online 21st June 2013

0959-4388/$ – see front matter, © 2013 The Authors. Published by Elsevier Ltd. All rights reserved.

**http://dx.doi.org/10.1016/j.conb.2013.05.004**

## Introduction

The prevalence of neurodegenerative diseases is growing relentlessly as the population ages. Regardless of their origin, these diseases are characterised by the aberrant aggregation of otherwise soluble proteins. In the case of Alzheimer's disease (AD) these are extracellular plaques of Aβ and intracellular filaments of the microtubule-associated protein tau, whilst α-synuclein deposits typify Lewy pathology disorders, including Parkinson's disease (PD). Huntington's disease (HD) is caused by huntingtin protein carrying an expanded number of CAG repeats, whilst tauopathy, synucleinopathy and expanded tri-nucleotide-repeats are also associated with other neurodegenerative diseases. The specific brain and spinal cord regions affected lead to the characteristic clinical manifestations [1].

**Figure 1:**
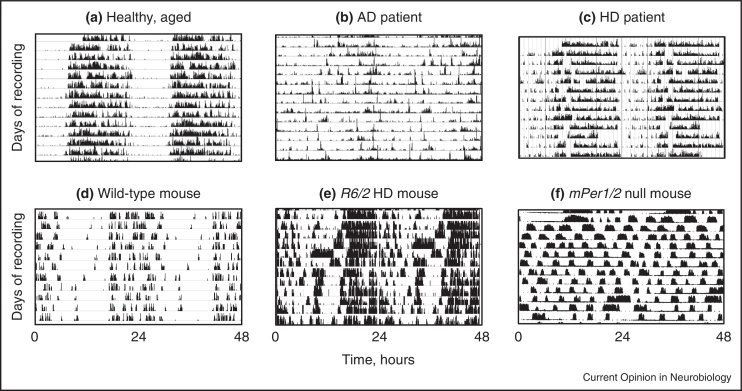
Daily cycles of rest, sleep and activity are disturbed in human neurodegenerative diseases and animal models thereof. Double-plotted actograms in humans and wheel-running behaviour in mice reveal stable behavioural patterns in unaffected individuals **(a, d)**, but severe disturbances in Alzheimer's disease and Huntington's disease **(b, c)**, the R6/2 mouse model of Huntington's disease **(e)** and a mouse lacking the circadian genes *mPer1* and *mPer2***(f)**. (a, b) from [3], (c) from [70], (d, e) from [6] and (f) unpublished from one of the authors (MHH).

One feature common to many neurodegenerative diseases is acceleration of the age-related disruption of the daily cycle of sleep and wakefulness [2–5]. This is evidenced both clinically and in animal models of disease (Figure 1) [6], and, because of the consequent disturbance of the sleep pattern of carers [7,8], is the principal reason for institutionalization of patients, with the attendant personal, economic and social costs. It is not yet clear whether these sleep disturbances arise from a defect in the core mechanisms that govern the timing of sleep and wakefulness (the suprachiasmatic nucleus or SCN), the nuclei that promote states of sleep (e.g. ventro-lateral pre-optic area (VLPO)) and wakefulness (e.g. tubero-mammillary nucleus (TMN) and locus coeruleus) or an inability of the diseased brain to sustain consolidated bouts of sleep and wakefulness, even though the timing pathways may be fully functional. For example, overall neurodegeneration within the SCN is seen in AD, and intriguingly the loss of different cell types within the SCN: arginine vasopressin (AVP) or neurotensin cells, is associated with differential effects, respectively, on activity rhythm fragmentation or activity (and temperature) rhythm amplitude [9]. On the other hand, neurodegenerative pathologies have also been observed in sleep-regulatory centres [7,10]. The pathobiological process resulting in AD may begin with mis-folded and abnormally hyper-phosphorylated tau protein located in the proximal axon of noradrenergic locus coeruleus projection neurons [11]. Subsequently, similar material fills the somato-dendritic compartment of these cells, resulting in their likely dysfunction. In separate experiments, it has been shown that the optogenetic activation of locus coeruleus neurons rapidly awoke mice that were asleep [12]. The prodrome of PD and dementia with Lewy bodies, which is characterised by neuropathological changes that precede parkinsonism and dementia, includes rapid eye movement (REM) sleep behaviour disorder, a parasomnia with dream-enacting behaviour, and loss of REM sleep atonia [13,14]. Lewy pathology is abundant in the lower brainstem, with only minimal damage in the substantia nigra [15^•^]. Because of the intimate relationship between circadian timing and sleep, and the widespread distribution of neuropathology, it is difficult, therefore, to disentangle cause and effect. For example, in the R6/2 mouse model of HD, circadian gene expression in the SCN was compromised *in vivo*, but when SCN tissue was cultured, circadian pacemaking was normal, suggesting that the dysfunction in the SCN was secondary to changes elsewhere in the brain [16].

Available evidence does not, therefore, allow identification of a single point of action or mechanism of effect to explain disordered sleep/wake patterns in neurodegenerative diseases. These observations have nevertheless spurred attempts to manage these diseases by applying a strict daily programme to impose more ordered sleep cycles upon patients and experimental mouse models. To date, these have met with mixed success. In a mouse model of HD, imposition of a more stable daily activity cycle by either pharmacological [16] or behavioural means [17] improved cognitive and metabolic functions, whereas daily treatment with melatonin and bright light had only a modest beneficial effect on cognitive function in institutionalized patients [18]. Regular bright light therapy can also alleviate depressive symptoms in elderly patients, and so may be a general pick-me-up [19]. At best, such approaches offer symptomatic relief.

The aim of this Opinion piece, therefore, is to consider the broader perspective provided by the latest developments in the cell biology of circadian clocks and neurodegenerative diseases. Regardless of clinical manifestations, the signature feature of disease is abnormal protein aggregation. *The fundamental issue, therefore, is the extent to which the circadian axis might impinge upon cellular pathology*. Importantly, this is the reverse of conventional approaches that have focused upon what neurodegeneration might do to circadian function.

## Circadian clocks co-ordinate cell biology in time

**Figure 2:**
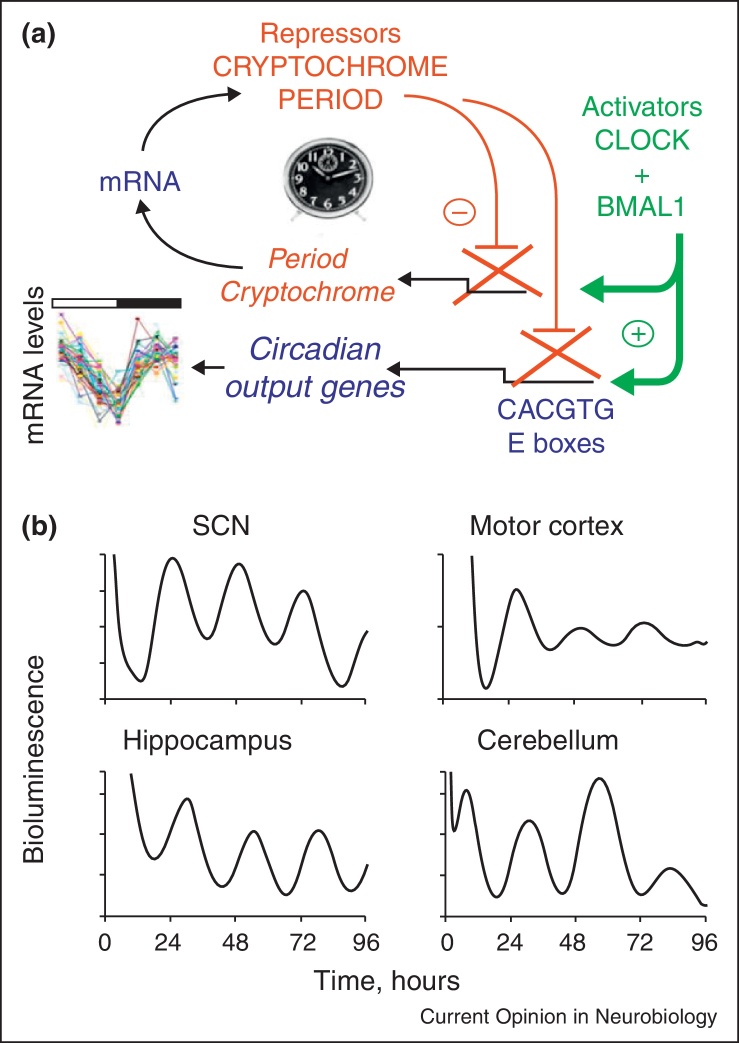
The circadian clock mechanism in mammals: a negative feedback oscillator distributed widely across the brain. **(a)** Schematic representation of the core negative feedback loop. Heterodimeric Clock/Bmal1 complexes transactivate *Period* and *Cryptochrome* during early circadian day via E-box regulatory sequences. Following translation and nuclear localisation of their own protein products, this leads to negative regulation of these genes during early circadian night. Subsequent degradation of Per and Cry during the night releases negative regulation and transactivation can start again, defining a new circadian day. Many downstream, clock-controlled genes also carry E-boxes and so are subject to alternate phases of activation and repression across the circadian cycle, thereby directing daily cascades of cellular function. **(b)** Distributed circadian clocks across the mammalian brain. Bioluminescence recordings from organotypic brain slice cultures derived from mPER2:LUC reporter mice demonstrate autonomous circadian molecular cycles, not only in the principal pacemaker of the SCN, but also in other brain regions. *In vivo*, these distributed clocks would be synchronised to the SCN pacemaker.

The detailed molecular genetics of circadian pacemaking are covered elsewhere (Hardin, this issue). In summary, the mammalian clock consists of a delayed feedback loop in which the circadian clock genes *Per* and *Cry* are negatively regulated by their cognate protein products on a daily basis (Figure 2a). This mechanism is recapitulated in most, if not all, cells and is understood at atomic resolution [20^•^,21^•^]. By virtue of its innervation by the retina, the interneuronal signalling that maintains its circuit-level pacemaking and its efferent connections, the SCN is the principal pacemaker. Importantly, other brain regions and peripheral tissues also contain local circadian clocks (Figure 2b) and the critical function of the SCN is that, via its regulation of neural, behavioural and endocrine outputs, it maintains circadian order across brain and body (Menaker, this issue). The importance of this order is that it establishes daily rhythms of metabolism and behaviour that adapt the individual to the day–night cycle and that it segregates incompatible biochemical processes in time. At the level of sleep, it is a given that effective sleep requires absolute synchrony between distributed clocks in the brain. Sleep and wakefulness are integrated, global states across the central nervous system that would be compromised by temporal disorder between different regions. The SCN clock synchronises local brain clocks to maintain this order.

Evidence of the deep nature of circadian temporal programming is provided by transcriptomic [22^•^,23^•^] and proteomic analyses [24^•^,25^•^] of liver, SCN and other tissues, which have shown that 5–10% of a local transcriptome is clock-regulated. In neurons, such a circadian programme, controlled by local clocks, will regulate synaptic, trophic and other processes, leading to systematic changes in the function of the neuron coincident with the SCN-determined cycle of wakefulness and sleep. Moreover, these local programmes are subject to alteration by sleep deprivation (Cirelli, this issue). The important point is that the cell biology of neurodegenerative aggregation very likely proceeds in the context of circadian modulation of some, perhaps many, of the key processes within the affected neuron and its surroundings. Although there has been some basic success in analysing the role of clock genes in sleep (Franken, this issue), compelling evidence for a contribution of clock gene dysfunction to neurodegenerative diseases and sleep disorders is lacking [26–28]. To explore this further, therefore, it is necessary to map out the generic events driving aggregation and to identify likely mechanisms for their circadian modulation. Are there circadian clock-dependent checkpoints in the mechanisms of neurodegenerative aggregation that might provide novel routes to therapy?

## The canonical signature of neurodegeneration is protein aggregation

**Figure 3:**
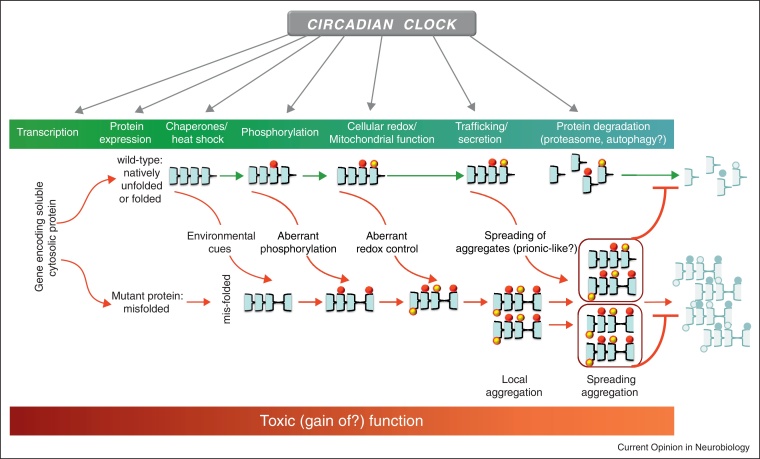
Circadian influences on neurodegenerative protein aggregation. Schematic model to highlight possible intersections between the circadian clock and mechanisms underpinning neurodegenerative protein aggregation. The life-history of benign cytosolic proteins is mapped in green as a series incorporating gene expression, folding, post-translational modifications (here represented by phosphorylation), oxidation and, ultimately, clearance through proteasomal degradation and autophagy. Pro-neurodegenerative mechanisms have been identified at every stage, leading to the aggregation of otherwise soluble proteins and their spreading through prion-like mechanisms. The origins of neurotoxicity are unclear, but presumably involve a gain of toxic function. Significantly, many, if not most, of these physiological and pro-neurodegenerative mechanisms are under circadian regulation. Thus, in addition to neurodegenerative diseases affecting the circadian clock, the circadian clock has the potential to promote (or hinder) disease progression.

Proof of the causative role of particular proteins in AD, PD, HD and other neurodegenerative diseases is provided by familial cases of disease that carry dominantly inherited mutations in the relevant genes [1]. Typically, the mis-folded proteins form highly ordered filamentous inclusions with a core region of cross-β-conformation. With the exception of HD and other tri-nucleotide repeat diseases, most cases of neurodegenerative disease occur in the absence of causative mutations, with protein mis-folding arising from stochastic, environmental and likely multigenic causes. This raises the question of how it is that the wild-type versions of these otherwise soluble proteins start to aggregate and kill neurons? Recent studies highlight major areas of interest (outlined in lower part of Figure 3), the first being the identity of the toxic factor(s). The progressive accumulation of large aggregates points inevitably to their causal involvement. An alternative view, however, is that toxicity arises from soluble intermediates or oligomeric forms, and that the end-point aggregates simply represent the nervous system's attempts to sequester proteins it is unable to dispose of by standard proteasomal and autophagic routes. Indeed, perhaps compromised function of the proteasome and autophagy arising from such intermediates may be the principal toxic insult to the cell (see below). In addition, there may be interactions between pathogenic proteins. For example, tau protein may be an arbiter of the pathological effects of Aβ in AD [29]. The origin of toxicity, however, is unclear, although possibilities include disordered synaptic signalling and disruption of particular organelles, for example mitochondria (see below).

But how might mis-folding occur and propagate in the first place? Recent attention has focused on the putative prion-like behaviour of abnormal protein aggregates. Until recently, aggregation was thought to be a cell-autonomous process, with aggregates forming independently in neurons and glia, and this may well be the case for the mutant proteins in familial cases. A series of recent studies has shown, however, that when aggregates were injected into the brains of recipient animals, they triggered aggregation of the cognate soluble protein in the host [1]. Significantly, in the case of tau, immunodepletion of the protein before injection prevented transmission, indicative of tau-mediated intercellular propagation of [30]. Similarly, the form of amyloid-β, can exhibit prion-like seeding of neurodegeneration in mouse brain that is dependent upon tau [31^•^]. In the clinical context of PD, α-synuclein-containing Lewy body pathology has been reported to be transmitted from the brains of patients into the foetal dopaminergic grafts they received as therapy [32–34]. A view is developing, therefore, that just as mis-folded prion protein is able to convert normal soluble protein to the mis-folded state, then so might neurodegenerative aggregates also spread by intercellular transmission.

The third point of interest is the potential contribution to neurodegeneration from reactive oxygen species (ROS) within a cell, and how they may trigger mis-handling of wild-type protein. Failure to control the oxidation state of proteins can arise from mitochondrial disease and compromised antioxidant function. Most directly, mutations of the antioxidant superoxide dismutase 1 (SOD1) cause amyotrophic lateral sclerosis (ALS), although this may reflect effects on aggregation rather than oxidation state. Nevertheless, more general associations have been drawn between proteins implicated in familial neurodegenerative diseases, including Parkin and α-synuclein, and mitochondrial function [35]. The two lines of potential causality: ROS causes mis-folding and aggregation, and aggregate or soluble proteins impair the ability of the cell to handle ROS, have yet to be disentangled.

A final emerging theme is that of autophagy. Under normal circumstances autophagosomes accumulate unwanted, damaged proteins and organelles and on merging with lysosomes, degrade them for export or re-cycling. Compromise of autophagy will aggravate ROS production by damaged mitochondria, and when induced by pharmacological or genetic means, aggravate the neurodegenerative condition in cell-based and animal models [36]. On the other hand, facilitation of autophagy can attenuate protein aggregation and neurodegeneration [37]. A more complex interplay between autophagy and neurodegeneration, however, has been revealed by the demonstration that both soluble and aggregated α-synucleins impair autophagy [38,39], again raising questions of cause and effect. If it is the general case that specific aggregate-prone proteins can disrupt the proteostasis of the entire cell [40], then the circadian clock, via its widespread influence upon the proteome [24^•^], may well be able to aggravate or attenuate aggregation-induced autophagy and susceptibility to mitochondrial dysfunction and cellular apoptosis.

## Where might clock-relevant processes intervene in protein aggregation and its consequences?

The pervasive influence of the circadian system on normal proteostasis and pro-neurodegenerative processes is outlined in Figure 3. The most immediate way for the clock to influence neurodegeneration is by circadian control over the expression of pro-neurodegenerative factors. This has been reported for several relevant genes in various rodent tissues [41^•^,42]. For example, presenilin-2, which regulates the cleavage of amyloid precursor protein (APP), is a direct target of CLOCK/BMAL1 transcriptional regulation, and is expressed with a strong circadian rhythm. TDP-43 is the principal pathogenic protein in tau-negative fronto-temporal dementia (FTLD-TDP) and in many forms of ALS, and at the transcript level is very highly circadian in mouse brain, as well as in liver and kidney. Similarly, α-synuclein and γ-synuclein are rhythmic in various tissues and the synuclein-interacting protein synphilin shows a strong circadian cycle in brain. This may be of direct relevance for disease, since the overexpression of wild-type α-synuclein can give rise to PD and dementia with Lewy bodies [43–45]. Genome-wide association studies have established that modest overexpression of α-synuclein may be a risk factor for idiopathic PD [46,47]. The same may also be true of multiple system atrophy (MSA), another synucleinopathy [48]. Similarly, mutations in *FUS* are associated with forms of FTD and ALS and again this gene is expressed with a very strong circadian rhythm in adrenal gland, liver and brain. At a transcriptional level, therefore, clock mechanisms may contribute to pro-neurodegenerative processes by elevating expression levels of the naturally occurring form of the protein on a recurrent daily basis, incurring progressive risk throughout the lifespan.

At the protein level, a healthy cell will progress through a daily cycle of alternating metabolic states directed by the circadian system, with proteins synthesised and degraded and organelles remodelled to sustain time-dependent cellular activities. Both the proteasomal and autophagocytic pathways play pivotal roles in this, releasing nutrients for re-cycling during periods of fasting and removing damaged or unnecessary organelles. In the liver and other tissues *in vivo* and in primary hepatocytes *in vitro*, this timely progression is underpinned by the circadian control of autophagy [49,50^•^]. Pivotal to this temporal control in the liver is circadian expression of the transcription factor C/EBPβ that in turn directs the circadian expression of a range of autophagy genes. It is not clear whether a comparable pathway exists in neurons, although *prima facie* there is no reason to believe something similar is not expressed, given the widespread incidence of autophagy rhythms in peripheral tissues and the expression of various C/EBP family members in the brain. Circadian modulation of autophagocytic capacity would establish particular phases of day or night when the neurons are more susceptible to aggregation and mitochondrial dysfunction, and potentially this would be exacerbated by circadian and/or sleep disturbance which would reduce the daily peak capacity for autophagy. In a similar vein, there is tight, reciprocal interplay between the circadian clock and the proteasomal machinery. First, genetic [51] or pharmacological [52] manipulations that compromise ubiquitination and proteasomal degradation of clock proteins impede the timely progress of the feedback loops, slowing the pacemaker and its dependent metabolic rhythms. Second, in the liver, a significant number of genes coding for chaperonins and elements of the ubiquitin/proteasome pathway are under circadian regulation (F box-containing proteins, ubiquitin-like proteins, ubiquitin-conjugating enzymes, ubiquitin fusion degradation-like proteins and proteasomal subunits) [23^•^]. As with autophagy, the capacity of cells to quality-control, degrade and clear unwanted proteins varies across the circadian cycle, thereby limiting the ability of the cell to deal with mis-folded monomers, toxic intermediates and aggregates alike. Disturbance of this circadian programme in neurons would enhance their susceptibility to a pre-existing neurodegenerative state.

A significant source of damage to pro-neurodegenerative proteins arises from oxidative stress, and circadian influences on the redox state of a cell are manifest in several ways. First, as with autophagy and proteasomal pathways, transcriptomic [22^•^,23^•^] and proteomic analyses [24^•^,25^•^] have shown that the expression of oxidative metabolic enzymes and antioxidant factors is modulated by the circadian system in both liver and brain. The ability of cells to deal with the consequences of dysfunctional mitochondria is therefore clock-gated. Mice deficient in the transcription factor BMAL1, an essential component of the mammalian clock, have reduced lifespans and display signs of premature aging in some non-neuronal tissues [53]. The phenotype of premature aging correlated with increased levels of reactive oxygen species. It will be interesting to see what happens in the brain of these animals and what effect the lack of BMAL1 has in existing models of human neurodegenerative diseases. Of course, the deficits may not be exclusively clock-dependent, insofar as BMAL1 may act in a non-circadian manner. Nevertheless, circadian disturbance from other sources may have an impact upon BMAL1 expression in its role as a clock component, and thereby affect its non-circadian actions. A second aspect arises from recent studies of circadian redox rhythms, evidenced by superoxidation of peroxiredoxins. In liver, these ubiquitous antioxidant proteins show high amplitude circadian cycles of transcription, protein abundance and post-translational modification [24^•^,25^•^]. Astonishingly, and counter to the transcriptional feedback loop model of the mammalian clock, circadian rhythms of post-translational superoxidation persist in anucleate erythrocytes, incapable of transcription. Comparable rhythms are also evident in peripheral tissues and in brain [54] and appear to be an ancient and deeply embedded feature of eukaryotic cells. The rhythm of superoxidation is a clear demonstration of circadian variation of the redox state of cells, and it has obvious implications for the progress of pro-neurodegenerative protein damage.

An additional post-translational modification implicated in neurodegeneration is phosphorylation, which can vary between soluble and aggregated states. Daily changes in the phosphorylation of circadian proteins are tightly regulated in order to control properties such as intracellular localisation, oligomerisation and degradation and *prima facie* similar programmes may be anticipated for at least some of the proteins that are prone to mis-folding in neurodegeneration. The abnormal hyper-phosphorylation of tau is an early and invariant feature of human tauopathies. Its increased phosphorylation is not necessarily detrimental, since it occurs reversibly during foetal brain development, hibernation and hypothermia [55–58]. The effect of hypothermia is the result of an exponential decrease in the activity of protein phosphatase 2A (PP2A), the major tau phosphatase in brain, and also a clock-relevant phosphatase [59] expressed with a circadian rhythm in SCN [41^•^]. Reductions in both PP2A expression and activity have been reported in AD brain [60,61]. Although it remains to be seen if different mechanisms underlie physiological and pathological tau phosphorylation, circadian mechanisms may influence tau phosphorylation through their control over the daily rhythm of core body temperature: a potent synchroniser of peripheral clocks [62].

As for possible interactions between clocks and prion-like propagation, there is only limited evidence. Disturbed sleep is a feature of prion diseases [63], not least fatal familial insomnia [64], and the absence of prion protein in mice alters sleeping patterns [65]. As for reciprocal effects, however, little is known, although circadian expression of mRNA encoding prion protein has been reported in rat brain [66]. In the absence of knowledge as to how prion-like aggregates may transmit pathology, it is difficult to divine circadian modulation of the underlying mechanisms [1], other than to highlight the strong circadian modulation of the expression of synaptic vesicle-cycling proteins in brain that would influence opportunities for exocytosis and endocytosis that could underlie prion-like intercellular transmission [25^•^]. Nevertheless, an intriguing relationship between sleep and Aβ accumulation has been described in mice transgenic for human mutant APP [67], with increased levels of Aβ in the interstitial fluid of the hippocampus during wakefulness. A comparable daily cycle of Aβ was also noted in human CSF. In preclinical AD, sleep quality deteriorated upon Aβ deposition, before cognitive symptoms [68^•^], so did Aβ plaques or sleep trouble develop first? In mice transgenic for human mutant APP, Aβ deposition preceded sleep abnormalities and the removal of plaques restored normal circadian rhythms [69^•^]. Conversely, sleep deprivation increased the levels of Aβ in the interstitial fluid of transgenic mice. These findings suggest that sleep disturbances and Aβ may influence each other through a positive feedback loop. Thus, the circadian sleep–wake cycle may contribute to the pathogenesis of AD, and perhaps other neurodegenerative diseases.

## Conclusion

It is well established that neurodegenerative diseases compromise sleep and circadian clock functions. But, whilst clock disturbance is not the primary cause of neurodegeneration, it is clear that as the disease progresses and impairs clock/sleep functions, this will enhance the brain's susceptibility to the pathology and aggravate its progression. Moreover, it is possible to identify several points in disease progression where exploitation of circadian principles could offer novel therapeutic approaches. Thus, dealing with and manipulating clock/sleep disturbances in neurodegenerative disease is not solely a symptomatic treatment: rather it may provide an important and novel avenue by which to ameliorate progress of the disease.

## References and recommended reading

Papers of particular interest, published within the period of review, have been highlighted as:

• of special interest
